# Magnitude of Anemia and Associated Factors among Pediatric HIV/AIDS Patients Attending Zewditu Memorial Hospital ART Clinic, Addis Ababa, Ethiopia

**DOI:** 10.1155/2015/479329

**Published:** 2015-03-24

**Authors:** Hylemariam Mihiretie, Bineyam Taye, Aster Tsegaye

**Affiliations:** ^1^Department of Medical Laboratory Sciences, Faculty of Medical and Health Sciences, Wollega University, P.O. Box 395, Nekemte, Ethiopia; ^2^Department of Medical Laboratory Sciences, School of Allied Health Sciences, College of Health Science, Addis Ababa University, P.O. Box 1176, Addis Ababa, Ethiopia

## Abstract

*Background*. Anemia is one of the most commonly observed hematological abnormalities and an independent prognostic marker of HIV disease. The aim of this study was to determine the magnitude of anemia and associated factors among pediatric HIV/AIDS patients attending Zewditu Memorial Hospital (ZMH) ART Clinic in Addis Ababa, Ethiopia. *Methods*. A cross-sectional study was conducted among pediatric HIV/AIDS patients of Zewditu Memorial Hospital (ZMH) between August 05, 2013, and November 25, 2013. A total of 180 children were selected consecutively. Stool specimen was collected and processed. A structured questionnaire was used to collect data on sociodemographic characteristics and associated risk factors. Data were entered into EpiData 3.1.1. and were analyzed using SPSS version 16 software. Logistic regressions were applied to assess any association between explanatory factors and outcome variables. *Results*. The total prevalence of anemia was 22.2% where 21 (52.5%), 17 (42.5%), and 2 (5.0%) patients had mild, moderate, and severe anemia. There was a significant increase in severity and prevalence of anemia in those with CD4+ T cell counts below 350 cells/*μ*L (*P* < 0.05). Having intestinal parasitic infections (AOR = 2.7, 95% CI, 1.1–7.2), having lower CD4+ T cell count (AOR = 3.8, 95% CI, 1.6–9.4), and being HAART naïve (AOR = 2.3, 95% CI, 1.6–9.4) were identified as significant predictors of anemia. *Conclusion*. Anemia was more prevalent and severe in patients with low CD4+ T cell counts, patients infected with intestinal parasites/helminthes, and HAART naïve patients. Therefore, public health measures and regular follow-up are necessary to prevent anemia.

## 1. Background

Hematological complications have been documented to be the second most common cause of morbidity and mortality in HIV positive persons [1]. Anemia, one of the commonest hematological complications with HIV infection, refers to a condition in which the hemoglobin content of the blood is lower than normal for a person's age, gender, and environment, resulting in the oxygen carrying capacity of the blood being reduced [2]. Anemia is a common feature of HIV infection, occurring in approximately 35% of patients who initiate antiretroviral treatment (ART) in Europe and North America [3].

In HIV-infected patients, anemia may be caused by nutrient deficiencies (iron, folic acid, and vitamin B12), sickle cell disease, HIV/AIDS itself, malaria, hookworm, and other infections. Other mechanisms for HIV-associated anemia, although uncommon, include autoimmune destruction of erythrocytes [4]. Direct infection of marrow precursor cells [5] has been hypothesized but not proven. The incidence of anemia ranges from 10% in people who have no HIV symptoms to 92% in individuals who have advanced AIDS [6]. Anemia has been reported as a very common complication of pediatric HIV infection, associated with a poor prognosis [7]. It has been identified as one of the predictors of early mortality in a cohort of HIV-infected children receiving HAART [8].

In established HIV infection, lower hemoglobin levels have been shown to correlate with decreasing CD4+ T cell counts which is supported by many studies demonstrating an association between anemia during established infection and a faster progression of AIDS as well as death. Therefore, interventions (like HAART administration) to prevent anemia may lead to improved health and survival potential of HIV-infected persons [9]. Anemia in children can be caused by iron deficiency and by health factors such as parasite infections or other causes. School children carry the heaviest burden of intestinal parasitic infection and anemia [10].

While there is a wide variation in the prevalence of anemia among HIV/AIDS patients in different studies all over the world, there is paucity of information on the prevalence and associated risk factors of anemia among pediatric HIV patients in Ethiopia. Pediatric ART started in the country relatively late. This study, therefore, aims to evaluate the magnitude of anemia among pediatric HIV positive patients based on age, gender, HAART status, intestinal parasitic infection, and CD4+ T cell levels.

## 2. Methods

### 2.1. Study Setting and Context

A comparative cross-sectional study was conducted in Zewditu Memorial Hospital (ZMH), Addis Ababa, Ethiopia, between August 05 and November 25, 2013. This hospital was selected due to the presence of large numbers of pediatric HIV patients under follow-up care and it is a model ART center as well. The hospital provides many health care services including pediatric HIV testing, counseling, and ART.

### 2.2. Study Population and Data Collection

One hundred eighty (180) pediatric HIV/AIDS patients (age less than 18 years) were enrolled consecutively. Guardians of patients and those children above 12 years of age were informed about the objective of the study. Then stool specimens were collected from each patient after getting written consent. Structured questionnaire was used to assess independent variables. Complete blood count and CD4+ T cell count, from EDTA whole blood using Cell-Dyn 1800 and FACScalibur, respectively, are routinely performed for patients on follow-up visits in ZMH. Accordingly, CD4+ T cell count and hemoglobin level were taken simultaneously with stool specimen collection. Therefore, no blood specimen was collected for the purpose of this study.

### 2.3. Specimen Collection and Processing

EDTA anticoagulated whole blood was run on CellDyn 1800 and FACSCalibur to determine hemoglobin level and CD4+ T cell count, respectively. Immunosuppression and anemia were defined based on WHO criteria [11] as follows—*mild anemia*: hemoglobin level between 10 and 10.9 g/dL for under 5 and between 11 and 11.9 g/dL for under 18 years of age children;* moderate anemia*: hemoglobin level between 7.0 and 9.9 g/dL for under 5 and between 8.0 and 10.9 g/dL for under 18 years of age children;* severe anemia*: hemoglobin level <7.0 g/dL for under 5 and <8.0 g/dL for under 18 years of age children;* mild immunosuppression*: CD4+T lymphocyte counts between 350 and 499 cells/*μ*L for under 18 or between 25 and 35% for under 5 years of age children [12];* advanced immunosuppression*: CD4+ T lymphocyte counts between 200 and 349 cells/*μ*L for under 18 or between 15 and 25% for under 5 years of age children [12];* severe immunosuppression*: CD4+ T cell count <200 cells/*μ*L for under 18 or less than 15% for under 5 years of age children [12]. Pediatric refers to children less than 18 years of age.* Magnitude of anemia* refers to severity and prevalence of anemia.

For parasitological analysis, a single stool specimen was collected from each patient using clean, dry, leak proof, and wide-mouthed caps. Direct wet mount, Formol-Ether concentration, and modified Zhiehl-Neelson staining techniques were applied to detect intestinal parasites microscopically. A small portion of the stool specimen was also preserved in 10% formalin to repeat tests whenever required and further analysis [13, 14].

### 2.4. Statistical Analysis

The data were cleaned, coded, and double-entered using EpiData version 3.1.1. and SPSS software version 16 (SPSS INC, Chicago, IL, USA) was used for data entry and analysis. Binary logistic regression was used to determine the association between anemia and demographic and clinical variables. Multiple logistic regressions were used to control the confounding factors. *P* values less than 0.05 were taken as statistically significant.

### 2.5. Ethical Considerations

The study protocol was ethically reviewed and approved by the Departmental Research and Ethical Committee of Addis Ababa University, Department of Medical Laboratory Sciences, and Addis Ababa Health Bureau. Then the Health Bureau sent a letter informing the hospital administrators about the study and hence permission was obtained from Zewditu Memorial Hospital. Data were collected after obtaining written consent from parents/guardians and confidentiality was maintained throughout the study by using codes. The positive results were timely reported to the clinicians for appropriate interventions.

## 3. Results

### 3.1. Sociodemographic Characteristics of the Participants

A total of 180 study participants were enrolled. Ninety-eight (54.4%) of them were males and 158 (87.8%) of them were urban residents. The mean age of the participants was 11 ± 3.2 and the median was 11 years ranging from 0.3 to 17 years. Majority (83.9%, 151/180) of the participants were at primary school level (Table 1).

### 3.2. Magnitude of Anemia and Associated Factors

The total prevalence of anemia was 22.2% (40/180) (Table 1). As summarized in the table, the prevalence of anemia was higher in females (24.4%) and rural residents (41%). Moreover, anemia showed higher incidence in patients aged 6–11 years (25%) and in primary school children (21.2%). When anemia was characterized by severity, mild, moderate, and severe anemia account for 21 (52.5%), 17 (42.5%), and 2 (5.0%) patients, respectively (Table 2).

Among anemic patients, 30% of males had mild anemia while 32.5% of urban residents were moderately anemic. Severe anemia was absent in patients classified under WHO HIV/AIDS stages I and II. HAART experienced patients had higher incidence of moderate anemia (12.5%) unlike HAART naïve patients who had the highest incidence of mild anemia (45%) (Table 2).

**Figure 1:**
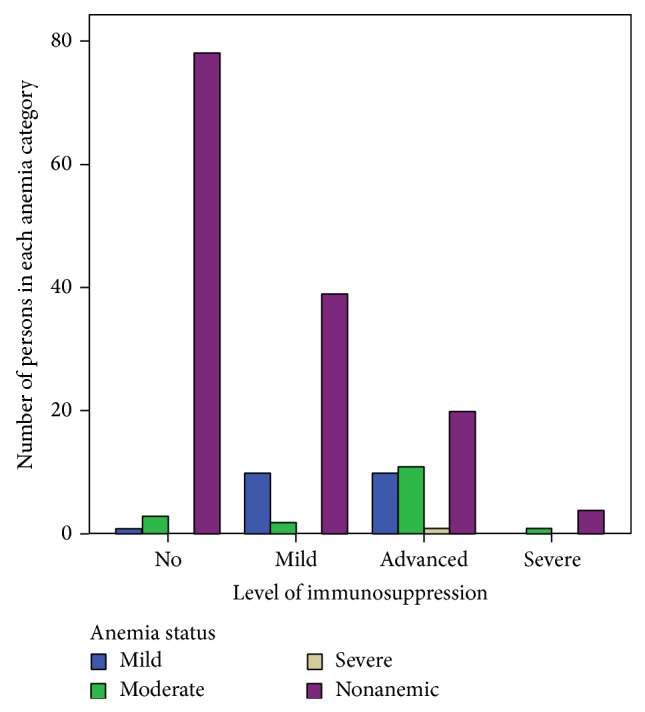
Distribution of anemia status by level of immunosuppression in Zewditu Memorial Hospital, from August 5, 2013, to November 25, 2013, Addis Ababa, Ethiopia.

More than half (54.8%) of those with advanced immunosuppression were anemic and severe anemia was also more prevalent in these groups (Figure 1). Figure 1 also shows that anemia was more prevalent in patients with mild immunosuppression. Both moderate and severe anemia showed higher prevalence in advanced immunosuppression while mild anemia was high in patients with mild immunosuppression.

Twenty-four (60%) of all anemic patients had CD4+ T cell count below 350 cells/*μ*L. Seventy-nine (43.9%) of the participants were HAART experienced patients. Among 40 anemic patients, 32 (80%) of them were HAART naïve. Mild (17.8%) and moderate (11.8%) anemia were more prevalent in HAART naïve patients (Table 2) and anemia was comparatively more prevalent and severe in HAART naïve patients (31.7%) (*P* < 0.05). Majority of the study participants, 123 (68.3%), were at WHO HIV/AIDS clinical stage II followed by clinical stage I (19.4%). Anemia was more prevalent in WHO clinical stages II and III patients, even though these stages were not independent risk factors to cause anemia (*P* > 0.05). Among 40 anemic patients, 28 (70%) were infected with intestinal parasites. Anemia significantly increased in those patients infected with intestinal parasites (*P* < 0.05) (Table 3).

In binary logistic regression, gender and age group do not show any significant association with anemia (*P* > 0.05), but WHO HIV/AIDS stage II (COR, 95% CI: 0.5 (0.1, 0.92), *P* < 0.05), absence of HAART (COR, 95% CI: 2.1 (1.1, 2.7), *P* < 0.01), rural residence (COR, 95% CI: 0.4 (0.1, 0.9), *P* < 0.05), primary school (COR, 95% CI: 3.2 (1.1, 10), *P* < 0.05), CD4 T cell count < 350 cells/*μ*L (COR, 95% CI: 2.5 (1.01, 4.1), *P* < 0.01), and infection with intestinal parasites (COR, 95% CI: 4.5 (1.2, 5.3), *P* < 0.01) showed significant association with the presence of anemia (Table 3).

After being adjusted with multinomial logistic regression, only absence of HAART (AOR, 95% CI: 2.3 (1.3, 4.7), *P* < 0.05), low CD4 T cell count <350 cells/*μ*L (AOR, 95% CI: 3.8 (1.6, 9.4), *P* < 0.05), and infection with intestinal parasites (AOR, 95% CI: 2.7 (1.1, 7.2), *P* < 0.05) were significantly associated with anemia (Table 3). Therefore, pediatric HIV/AIDS patients without HAART, infected with intestinal parasites, and having low CD4+ T cell counts had 2.3, 2.7, and 3.8 times more likelihood of being anemic compared to their counterparts, respectively (Table 3).

One-way ANOVA analysis showed that there was significant difference in mean hemoglobin concentration between and within the groups of presence of intestinal parasites, CD4+ T cell category, and HAART status (Table 4).

## 4. Discussion

Anemia in children can be caused by iron deficiency and by health factors such as parasite infections. Genetic disorders such as the hemoglobinopathies and thalassemias are also implicated in some parts of the world. School children carry the heaviest burden of intestinal parasitic infection and anemia [10].

The observed prevalence of anemia in this study (22.2%) is very low compared to a study done in Tanzania (77.4%) [15], Northeastern Nigeria (57.5%) [16], Gondar (70.1%) [17], in Northwest Ethiopia (35%) [18], and Ghana (46%) [19] but is comparable to a study done in Jimma (21.9%) [20] and Southwest Ethiopia (23.1%) [21]. The possible explanation to this low prevalence might be due to low prevalence of helminthes in this study, better follow-up, and better awareness of participants about anemia and being urban residents.

The respective 21 (52.5%) and 17 (42.5%) mild and moderate anemia observed in this study are higher than a study in Tanzania [15] where mild and moderate anemia account for 28.4% and 32.1%, respectively. Furthermore severe anemia (5.0%) is higher than a study in North West Ethiopia (1.3%) [18]. This difference might be due to geographic difference as most of them used more than one study area, age difference as our participants are pediatric and anemia can be caused by different factors including nutrition in children, and sample size difference as our sample size is smaller than the previous studies which may increase the prevalence. Unlike studies from North West Ethiopia [18] and Tanzania [15], anemia was not significantly associated with gender in the present study which might be due to comparable number of both genders participated in this study. Moreover, mild and moderate anemia were more prevalent in advanced immunosuppression which is in agreement with a study done in Jimma [20].

Similar to the previous studies done in North West Ethiopia [18], Ghana [19], Jimma [20], Addis Ababa [22], and Northeastern Nigeria [16], the prevalence of anemia was significantly associated with lower CD4+ T cell levels but different from a study in West Africa [23] where CD4+ T cell count was not significant predictor of anemia.

The prevalence of anemia was found to be higher among rural residents, even though residence was not an independent predictor of anemia unlike a study done in Jimma [20] where rural residence was associated with significantly increased anemia. This higher prevalence of anemia in rural residents might be due to the fact that those participants residing in rural areas might not have adequate information about nutrition and other factors that could cause anemia.

Intestinal parasitic infections, lower CD4+ T cell count, and being HAART naïve were identified as significant predictors of anemia. Patients with intestinal parasitic infections had 2.7 times more risk of developing anemia compared to those without infections (*P* = 0.048, 95% CI: 1.1–7.2). The risk of developing anemia in patients with low CD4 count was 3.8 times more than those with higher CD4 count of >350 cells/*μ*L (*P* = 0.03, 95% CI: 1.6–9.4). On the other hand HAART naïve patients had 2.3 times more likelihood of being anemic than their counterparts. This may indicate that the likelihood of anemia increases with immunologic deterioration and with the advancement of HIV-related disease in the absence of HAART. This result is comparable with a study done in Southwest Ethiopia [21].

## 5. Conclusion

The prevalence of anemia in pediatric patients was significantly high in this study. The prevalence and severity of anemia was significantly increased in HAART naïve patients, those infected by intestinal parasites, and in immunosuppressed HIV patients. In conclusion, this study has shown that HAART initiation, anthelminthic medication, and regular checkup of CD4 count might have benefit in reducing anemia in HIV positive pediatric patients. Therefore, further longitudinal studies with long-term follow-up are needed to explore more on the causes of anemia and the pattern of hemoglobin changes with associated factors in HIV positive persons in resource limited settings.

## Figures and Tables

**Table 1:** Sociodemographic characteristics of pediatric HIV/AIDS patients attending Zewditu Memorial Hospital from August 5, 2013, to November 25, 2013, Addis Ababa, Ethiopia (*N* = 180).

Variables	Anemia		Total *N* (%)
	Present *N* (%)	Absent *N* (%)	
Age group (years)			
<2	1 (50)	1 (50)	2 (1.1)
2–5	2 (33.3)	4 (66.7)	6 (3.3)
6–11	21 (25.3)	62 (74.7)	83 (46.1)
12–18	16 (18)	73 (82)	89 (49.5)
Sex			
Male	20 (20.4)	78 (79.6)	98 (54.4)
Female	20 (24.4)	62 (75.6)	82 (45.6)
Residence			
Urban	31 (19.6)	127 (80.4)	158 (87.8)
Rural	9 (41)	13 (59)	22 (12.2)
Education			
Did not begin	1 (25)	3 (75)	4 (2.2)
Kindergarten	1 (8.3)	11 (91.7)	12 (6.7)
Primary	32 (21.2)	119 (78.8)	151 (83.9)
Secondary	6 (46)	7 (54)	13 (7.2)
Total	**40 (22.2)**	**140 (77.8)**	**180 (100)**

**Table 2:** Frequency distribution of anemia status among anemic pediatric HIV/AIDS patients attending Zewditu Memorial Hospital from August 5, 2013, to November 25, 2013, Addis Ababa, Ethiopia (*N* = 40).

Variables	Mild *N* (%)	Moderate *N* (%)	Severe *N* (%)	Total
Age group (years)				
<2	0 (0)	1 (2.5)	0 (0)	1 (2.5)
2–5	2 (5)	0 (0)	0 (0)	2 (5)
6–11	12 (30)	7 (17.5)	2 (5)	21 (52.5)
12–18	7 (17.5)	9 (22.5)	0 (0)	16 (40)
Gender				
Male	12 (30)	8 (20)	0 (0)	20 (50)
Female	9 (22.5)	9 (22.5)	2 (5)	20 (50)
Education				
Did not begin	0 (0)	1 (2.5)	0 (0)	1 (2.5)
Kindergarten	1 (2.5)	0 (0)	0 (0)	1 (2.5)
Primary	18 (45)	12 (30)	2 (5)	32 (80)
Secondary	2 (5)	4 (10)	0 (0)	6 (15)
Residence				
Urban	18 (45)	13 (32.5)	0	31 (77.5)
Rural	3 (7.5)	4 (10)	2 (5)	9 (22.5)
HAART I				
Yes	3 (7.5)	5 (12.5)	0 (0)	8 (20)
No	18 (45)	12 (30)	2 (5)	32 (80)
WHO stage				
I	3 (7.5)	1 (2.5)	0 (0)	4 (10)
II	16 (40)	10 (25)	0 (0)	26 (65)
III	2 (5)	6 (15)	1 (2.5)	9 (22.5)
IV	0 (0)	0 (0)	1 (2.5)	1 (2.5)
Total	**21 (52.5)**	**17 (42.5)**	**2 (5)**	**40 (100)**

**Table 3:** Association of risk factors with anemia in pediatric HIV/AIDS patients attending Zewditu Memorial Hospital from August 5, 2013, to November 25, 2013, Addis Ababa, Ethiopia (*N* = 180).

Variables	Anemia		COR (95% CI)	*P*	AOR (95% CI)	*P*
	Present *N* (%)	Absent *N* (%)				
WHO S						
I	4 (10)	31 (22.2)	1		1	
II	27 (67.5)	96 (68.6)	0.5 (0.1, 0.92)	0.008^*^	0.3 (0.01, 1.1)	0.4
III	9 (22.5)	11 (7.8)	0.2 (0.04, 0.6)	0.2	1.2 (0.1, 3.2)	0.1
IV	0 (0)	2 (1.4)	0 (0, 0)	1.00	0.1 (0, 1.3)	0.9
HAART I						
Yes	8 (20)	71 (50.7)	1		1	
No	32 (80)	69 (49.3)	2.1 (1.1, 2.7)	0.001^*^	2.3 (1.3, 4.7)	0.048^*^
Residence						
Urban	31 (19.6)	127 (80.4)	1		1	
Rural	9 (41)	13 (59)	0.4 (0.1, 0.9)	0.03^*^	0.6 (0.2, 2.3)	0.5
Edu. s.						
NB	1 (25)	3 (75)	2.6 (0.2, 32)	0.5		
KG	1 (8.3)	11 (91.7)	9.4 (0.9, 96)	0.3		
Prim.	32 (21.2)	119 (78.8)	3.2 (1.1, 10)	0.05^*^	0.3 (0.06, 1.3)	0.09
Secon.	6 (46)	7 (54)	1		1	
CD4						
<350	24 (60)	24 (17)	2.5 (1.01, 4.1)	0.00^*^	3.8 (1.6, 9.4)	0.03^*^
>350	16 (40)	116 (83)	1		1	
IPs						
Present	28 (70)	40 (28.6)	4.5 (1.2, 5.3)	0.00^*^	2.7 (1.1, 7.2)	0.048^*^
Absent	12 (30)	100 (71.4)	1		1	
Age group						
<2	1 (50)	1 (50)	0.2 (0.01, 3.7)	0.3		
2–5	2 (33.3)	4 (66.7)	0.4 (0.07, 2.6)	0.36		
6–11	21 (25.3)	62 (74.7)	0.6 (0.3, 1.30)	0.25		
12–18	16 (18)	73 (82)	1			
Sex						
Male	20 (20.4)	78 (79.6)	1			
Female	20 (24.4)	62 (75.6)	0.8 (0.4, 1.6)	0.5		

^*^Significant at P value <0.05, AOR: adjusted odds ratio, COR: crude odds ratio, WHO S: WHO HIV/AIDS stage, HAART I: HAART initiation, P: P value, IPs: intestinal parasites, Edu. s: educational status, KG: kindergarten, Prim.: primary, Secon.: secondary, NB: did not begin, CD4: CD4 T cell count (cells/μL).

**Table 4:** One-way ANOVA analysis of hemoglobin concentration among pediatric HIV/AIDS patients attending Zewditu Memorial Hospital from August 5, 2013, to November 25, 2013, Addis Ababa, Ethiopia.

Variables	Mean square		*F*	*P* value
	Within groups	Between groups		
Gender	2.9	0.95	0.32	0.6
HAART status	2.7	59	25.6	0.04
CD4 category	2.6	71	29.2	0.02
Infection with intestinal parasites	2.5	88	35.8	0.00
